# Value-Creating Strategies in Dairy Farm Entrepreneurship: A Case Study in Northern Spain

**DOI:** 10.3390/ani11051396

**Published:** 2021-05-13

**Authors:** Antonio Alvarez, Beatriz García-Cornejo, José A. Pérez-Méndez, David Roibás

**Affiliations:** 1 Department of Economics, Faculty of Economics and Business Administration, University of Oviedo, Av. del Cristo s/n, 33006 Oviedo, Spain; alvarez@uniovi.es (A.A.); droibas@uniovi.es (D.R.); 2 Department of Accounting, Faculty of Economics and Business Administration, University of Oviedo, Av. del Cristo s/n, 33006 Oviedo, Spain; bgarciac@uniovi.es

**Keywords:** value-creating strategies, farm diversification, dairy, performance

## Abstract

**Simple Summary:**

The aim of this paper is to analyse the heterogeneity and the performance of the value-creating strategies applied by dairy farms in their diversification initiatives. This is a pertinent topic to study because the Common Agricultural Policy provides a role for value-added diversification strategies as an alternative to ensure the economic viability of small farms. Furthermore, the fostering of specialised policy initiatives requires empirical evidence on specific diversification strategies. Nevertheless, there are still few quantitative studies analysing the factors that influence the success of value-added initiatives in terms of their contribution to economic performance, which may be due to the difficulty of obtaining databases such as the one handled in this study. Our findings confirm that the strategies are associated with different combinations of contexts and entrepreneurial capabilities. In fact, the results of this research present practical implications for future entrepreneurs and for policymakers, contributing towards understanding how an adequate alignment between contextual factors and entrepreneurial capabilities can enable the implementation and sustainability of new business initiatives in rural areas. We also conclude that the diversification ventures of dairy farms are an appropriate element for guaranteeing the survival of certain local breeds, making their use profitable.

**Abstract:**

This paper explores different value-creating strategies (VCS) used by dairy farmers engaged in on-farm diversification ventures. In order to explicitly identify the informal strategies followed by 49 farmers in their value-added ventures, we applied a theoretically informed business model framework combining three dimensions: value proposition linked to local food, customer engagement via quality schemes and shorter supply chains, and the key capabilities of the entrepreneur. Using cluster analysis, four different types of VCS were identified and labelled as ‘Ecological’, ‘Single-product’, ‘Innovative’, and ‘Traditional’. Whilst we found that these strategies are influenced by contextual factors and the owner’s entrepreneurial skills, in general, we did not observe significant differences in performance between them. The results suggest that farmers respond entrepreneurially to sectorial changes adopting those VCS that tend to align with their entrepreneurial capabilities and context, thus enabling them to succeed with any of the strategies pursued. Hence, our work contributes towards clarifying the relationship between VCS, entrepreneurial capabilities, and context. This is important for farmers and policymakers because it reveals the diversity of farm management and the resilience of farm systems. As a result, the potential challenges for Single-product VCS and Traditional VCS are discussed.

## 1. Introduction

This study investigates the relationship between value-creating strategies (VCS), linked to contextual and personal factors, and economic performance in dairy farm diversification processes. The restructuring of the European agricultural sector resulting from globalisation and various Common Agricultural Policy (CAP) reforms has forced numerous farmers to deploy strategising and marketing skills, where the latter is required to adopt a stronger entrepreneurial orientation (EO) in order to guarantee the sustainability of their business [1]. Specifically, the European farm-to-fork strategy focuses on shorter supply chains in order to enhance the resilience of regional and local food systems, highlighting the role of geographical indications and organic production [2,3].

Regarding the EU dairy sector, CAP reforms since 2003 have led to a reduction in intervention prices, so the price received by the farmer is determined to a greater extent by market forces, becoming more in line with world prices. Although the Spanish production of cow’s milk has grown from 2010 to 2020 by 27%, especially since the abolition of the quota system in 2015, Spain continues to present a deficit, with national production covering 77.5% of domestic consumption in 2020 [4]. The main production of the Spanish dairy industry continues to be liquid milk (45% of raw milk at the beginning of 2021) [5,6], with store brands having great relevance in its marketing [7]. In Spain, most dairy farmers are focused on milk production as a commodity, limited by a low level of bargaining power in the food chain. In this context, farmers and the processing industry need to learn how to survive in a more market-oriented environment. Therefore, initiatives that seek to improve the productive and commercial efficiency of the different actors in the dairy sector are of great interest, especially when considering the new challenges they face such as those related to sustainability and resilience [8].

These changes in the dairy sector provide a role for value-added diversification strategies as an alternative for ensuring the economic viability of small farms [9]. This is a pressing need, as illustrated by the rapid abandonment of dairy farmers located in the four coastal regions of Northern Spain considered in this study (Asturias, Cantabria, Galicia, and the Basque Country). In particular, over the period 2010–2020, the number of dairy farmers in those regions fell from 17,980 to 9645 [10,11]. Abandonment of agricultural activity can also have social costs, such as rural depopulation, and negative environmental impacts [12,13]. In this context, VCS applied in diversification initiatives can play a relevant role to guarantee the sustainability (economic, social, and environmental) of the farms that adopt them.

A substantial body of literature exists on agricultural diversification [14,15,16] and farm diversification strategies [17,18], but empirical studies analysing the influence on farm economic performance are scarce [19,20,21,22]. Most studies consider diversification as a pool of income-earning opportunities complementing the core agricultural production, without distinguishing between different types of activities [23]. There are only a few studies on value-added initiatives and their impact on farm profitability, mainly from a qualitative perspective [9,24]. In relation to dairy farms, much of the research has focused on the profitability and efficiency of milk production [25,26,27]. However, a gap exists with respect to the study of the profitability of processing activities [28,29], and this is due to the difficulty in obtaining data and the problem associated with separating the value of transformation activities from the milk production results.

Existing studies show that more diversified farmers have stronger entrepreneurial identities and employ more complex business strategies than farmers who do not diversify [30,31]. However, there is a lack of knowledge regarding the heterogeneity of strategies pursued by farmers when seeking to add value to their primary production. Furthermore, mainstream entrepreneurship literature requires more focus on the context, as evidenced in recent rural entrepreneurship studies [32,33]. Context simultaneously provides individuals with entrepreneurial opportunities and sets boundaries for their actions; in other words, individuals may experience it as both an asset and a liability [34]. Therefore, the influence of external factors together with internal or personal factors should be considered when analysing the entrepreneurial actions of individuals [35,36].

Although previous studies have captured the different entrepreneurial approaches adopted by farmers (see [32] for a review), they do not usually perform a joint analysis of farmers’ personal characteristics and the particular context framing their ventures, thus overlooking how farmers combine their capabilities and conditions for strategy development purposes.

This paper contributes to the research regarding the strategies of micro-level entrepreneurial ventures in the rural sector [32,33,37,38], particularly in a small farm setting. We do this by identifying the VCS used by dairy farmers engaged in on-farm diversification. We employ a theoretically informed conceptual business model [39] in order to identify explicitly the informal strategies followed by dairy farmers in their value-added ventures. Given that most of these farmers do not adopt a formal strategic planning approach [40], our empirical research is based on the Business Model Canvas (BMC) approach [41] as a suitable conceptual framework to explore the ways in which farmers create value through new ventures [42]. We selected the BMC because of its conceptual clarity with respect to the business aspects of the diversification initiatives, as well as its holistic approach [39]. The BMC considers the key resources and activities for developing the value proposition delivered to consumers through different local products, quality schemes, milk mixtures from different animals (cows, goats, and sheep) and breeds, sales formats, and distribution channels/shorter supply chains. 

Accordingly, the aim of the present research is to analyse the heterogeneity and the performance of VCS used in dairy farms that diversify their activity by adding value to raw milk through the elaboration and sale of different dairy products (fresh milk, cheese, yogurt, and others). Considering the critical role of context on entrepreneurial activities [34,43], this paper attempts to answer the following research questions:

RQ1: What types of VCS are used by dairy farmers engaged in on-farm diversification?

RQ2: How are VCS associated with different contextual and personal factors?

RQ3: Are there differences in performance between VCS?

## 2. Materials and Methods

Our study focuses on the diversification ventures but not on the activity of the farm as a whole. Therefore, the data used in the empirical analysis refer fundamentally to these initiatives. 

### 2.1. Sample and Data Collection

Although, in the Spanish dairy sector, most of the production is sold directly to the processing industry, a certain number of farmers have embarked upon diversification strategies.

This study centres on the four regions of Northern Spain (Asturias, Cantabria, Galicia, and the Basque Country), where 79.3% of the dairy farmers and 55.1% of Spanish dairy production were located in 2020 [11]. We identified the dairy farms that process and sell value-added products. To do this we searched on the Internet and made enquiries to different agents of the dairy sector (cooperatives, advisors, producer organisations, regulatory organisms of the different product labels and organic agriculture, etc.). Collaboration was requested from the 80 farms identified, finally obtaining the effective participation of 49 entrepreneurial farmers through in-depth interviews. This allowed us to use original quantitative and qualitative data and carry out a fine-grained economic analysis of the value creation process in these ventures.

We designed a questionnaire for the purpose of collecting information via personal interviews. For its design, we reviewed the literature [14,16,19,37,38,44,45,46,47,48,49,50,51,52] and conducted exploratory interviews with a total of 25 experts in the dairy sector who worked in different fields related to farms: farm advisers (6), scientists (8), farm union staff (4), representatives from government (4), and rural development (3). These experts provided us with insights concerning the key factors defining the strategy for these types of ventures: value proposition, protected designation of origin (PDO) certification, organic label, sales structure by product types, sales channels, the number of product references, knowledge management, participative management, product innovation, and the use of networks.

Each of the 49 farmers was interviewed on their own farm, a process lasting two hours on average. The meetings included informal discussion prior to and following the formal interview, which were recorded and transcribed. Prompts were utilised to ensure the data collected covered the research objectives, but discussions were allowed to flow as freely as possible. The data collection through face-to-face, in-depth interviews allowed us to complement the study with narrative elements important in the strategic formulation. Additionally, we were able to identify the farmer’s motivations for diversifying as well as analysing the context in which the initiative was developed. 

The data, collected in 2012, refer to 2011. The dairy farms visited reported an average raw milk production volume of 518,000 litres, which represented 178% of the average production per dairy farm in Spain. However, there was a large dispersion in this variable between the different VCS identified. Table 1 shows descriptive statistics of the sample of diversification initiatives.

The average volume of processed milk per farm amounted to 238,548 litres; 87.2% of the milk used for the elaboration of different products was cow milk, and the remainder was goat or sheep milk. The average investment dedicated to the processing and marketing of dairy products reached a value of EUR 257,500 per farm, 76% of this being allocated to assets related to milk processing and the rest to the marketing and distribution of products. The average number of workers per farm dedicated to activities related to the processing and commercialisation of products was 3.1, and these workers accounted for 64% of employment in the sampled farms.

The average value of sales per farm was EUR 256,808. The average sales structure by product type indicated that cheese represented 54.1% of total sales, fresh milk 22.5%, yogurt 11.3%, and other products the remainder. The average sales structure in terms of marketing channels reflected sales to end consumers of 35.2%, with hospitality, shops, and large-scale distribution representing 21%, 30%, and 13.8% of sales, respectively. Products with an organic label accounted for an average of 26.8% of value-added venture sales, while sales with a PDO certification represented an average of 22.1% of sales per venture.

### 2.2. Conceptual Framework: Business Model Canvas to Value-Creating Analysis

Given that most of these farmers do not adopt a formal strategic planning approach [40], our empirical research is based on the Business Model Canvas (BMC) [41] as a conceptual framework to explore the ways in which farmers create value through new ventures. We found this framework well-suited for systematising our data, thereby facilitating the explicit identification of strategies used by farmers as well as permitting quantitative analysis. In short, we found it particularly helpful in the modelling process [39]. 

BMC is structured around four main areas: value proposition, customer, infrastructure management (key resources, key activities, and key partnerships), and financial aspects. We adapted the framework to the objectives of this study by choosing ten strategic and operational indicators for three areas: value proposition, customer, and key activities. Firstly, for each farm we measured the ten indicators to explore the different groups of VCS. Next, we analysed the differences in the performance indicators between these groups. We excluded the financial area because our objective was to identify groups in line with the strategic and operative factors that explain the value creation. If we included performance variables in the cluster analysis, the groups would be defined by these variables. Finally, the revenue model is a result of the operative and strategic model [42]. 

A value proposition can be defined as the “expression of the experience that a customer will receive from a supplier’s measurably value-creating offering” [44]. As such, it does not represent what the firm does. Instead, it is the value experience that the firm delivers. The farms that embark upon value-added diversification initiatives base their strategy on local food through industry differentiation. They seek to achieve a market niche integrated by consumers who demand traditional products requiring high welfare standards, environmental or otherwise [45,53,54,55]. 

The customer area is related to customer engagement, defining the targeted audience, the distribution channels, and the type of relationship the company maintains with their clients. The definition of the term ‘local food’ applied in this study refers to the quality dimension, as opposed to the geographical dimension, of the concept. Under this definition, the product is not necessarily consumed in the same region or locality of origin and “is identified and distinguished using product labels, certification systems and other production parameters such as artisanal, traditional, farm based, organic or natural to define and differentiate the quality of the specific product coming from a specific geographic area” [56]. Regarding distribution channels, typical direct marketing strategies or shorter supply chains include selling from the farm, farm stands, Internet/mail-order sales or farmer markets, as well as direct sales to restaurants, grocery stores, or institutions [45].

Regarding the key activities, the business model framework takes into account the internal capabilities that give rise to a competitive advantage. A firm’s competitive advantage may derive from either a better execution of certain activities within the value chain, from a better coordination of these activities, or from a better management of its networks [57]. Previous research on farm entrepreneurship has identified the factors that influence the performance of new initiatives [19,20,58,59]. The following critical capabilities in farms can be identified: knowledge management, participative management, innovation, and networks. 

Small firms, including farms, manage knowledge informally as part of their normal activities without the use of terminology and concepts of knowledge management [60]. This idea is supported by the author of [20], who finds that the integration of knowledge (acquired via training and the observation of similar experiences) is a critical factor in the development of products, production processes, and marketing in those agricultural farms embarking upon new initiatives.

Participative management is characterised by employees being involved and committed in the decision-making process, and this must be accompanied by a reward system linked to improving firm productivity and customer satisfaction [48,49,50].

For the dairy farms focused on value-added processing, innovation does not imply outlays of R&D, but instead it is implemented, for example, by increasing their portfolio with higher-value-added products or by differentiated packaging [61], or by the conservation of traditional production methods, which are often economically disadvantageous in terms of costs, but allow for higher prices due to the potential obtainment of PDO certification [62]. Previous research shows that an owner’s innovativeness has a positive influence on market orientation, innovation, and performance in small firms [63].

The use of networks facilitates access to information, resources, and markets. Its importance will be greater for small and rural enterprises, which face less favourable conditions in terms of location, information sources, market proximity, and the possibility to develop their own R&D [19]. Various studies have highlighted the important role played by the construction of formal (professional) and informal (social) networks in the success of new initiatives [20,64,65].

### 2.3. Measurements

Two types of variables were used: (1) factors extracted from a principal component analysis based on Likert-style questions (from 1 to 5) and (2) variables measured directly from interviews or calculated using the information gathered, as in the case of the economic indicators of costs and margins. 

Each construct or factor was measured through various items, using previous papers as a reference and, in some cases, adapting these to the characteristics of the dairy farms. The constructs used in the analysis and the references on which they are based are presented in Table 2. For their measurement, the farmers were asked to value the degree of implementation of the different practices over the past three years on a scale ranging from 1 ‘very little implementation’ to 5 ‘a lot of implementation’. In the same way, they ranked several motivations for the creation of the value-added venture, ranging from 1 ‘strongly disagree’ to 5 ‘strongly agree’. In order to measure the value proposition, interviewees were asked to value three aspects of their company as compared with the competition: (a) ability to make quality products, (b) the responsibility of the company with respect to the environment, and (c) customer satisfaction. Each item was measured on a scale ranging from 1 ‘very inferior to the competition’ to 5 ‘very superior to the competition’.

Regarding performance, we used a qualitative measure based on the subjective perception of the interviewees, as well as three quantitative measures determined using accounting data. The factor denominated ‘performance’ was measured using three items, so that farmers were asked what they considered to be the situation of their farm compared with competitors in terms of (a) optimisation of the investment in assets, (b) competitiveness of the farm, and (c) profitability of the farm. In order to measure each item a scale was used, ranging from 1 ‘very inferior to competitors’ to 5 ‘very superior to competitors’. The three quantitative variables based on accounting data were margin per litre, profit per litre, and value added per worker.

Entrepreneurial Orientation (EO) refers to a firm’s strategic orientation, reflecting the decision-making styles, practices, and methods that direct its activities [66]. EO is defined by three dimensions: innovativeness, proactiveness, and risk-taking [67,68]. We assessed the farmers’ EO through their narratives and variables based on several factors: product innovation, motivations to diversify, the strategic behaviour, and the capabilities of the farmer related to knowledge management and participative management. 

Innovativeness and product innovation reflects the firm’s tendency to embrace new ideas, favour change, and encourage experimentation [69]. Previous research found that EO has a positive influence on new product or process innovation [70]. 

The economic motivation behind agricultural diversification better reflects the willingness of the farmer to assume the risks of starting a business, with a higher risk of failure compared to social or lifestyle motivations [16].

Given that the proactiveness of EO most closely resembles the ideas suggested by Miles and Snow’s [71] prospector type [66], we used the Miles and Snow typology so that farmers would classify themselves either as prospectors, analysers, defenders, or reactors in line with each one’s strategic behaviour.

Previous research found that EO has a positive influence on the capability of an organisation to manage their knowledge [70,72]. On the other hand, while EO mobilises family members to search for new opportunities, the coordination provided by a participative strategy ensures the most effective vetting of those opportunities [73].

Additional information on the productive and commercial characteristics of the diversification initiatives has also been obtained.

### 2.4. Statistical Techniques

We used principal component analysis to obtain the constructs shown in Table 2 and cluster analysis in order to identify different VCS applied by dairy farmers in their diversification initiatives. Cluster analysis is a multivariate technique that classifies observations into homogenous groups with respect to some selection criterion. The cases in each cluster can be considered ‘similar’, while the different clusters are assumed to be ‘distinct’ from each other [74]. We used K-means clustering, a very popular technique, which is not without its problems due to some strong assumptions and limitations [75]. K-means clustering requires a priori specification of the number of clusters and assumes that all clusters are equally sized and have the same variances. The results vary depending on the choice of the variables used, which must be standardised, as well as being sensitive to the initial seeds and to the presence of outliers. These limitations do not preclude the use of K-means clustering but may affect the results of the analysis. This must be taken into account when interpreting the results, where the reasoned judgment of the researcher is also important [74].

The differences between VCS were first analysed using the Kruskal–Wallis test, which detects whether at least one of the VCS is different from the rest. Next, we used Dunn’s test to analyse the existence of significant differences between the specific pairs of the VCS. In the case of dummy variables, Pearson’s chi-square (χ2) test was used. We used the software IBM SPSS Statistics 24.

## 3. Results

### 3.1. Constructs Used in the Analysis

Table 2 shows that the Kaiser–Meyer–Olkin (KMO) and Sphericity tests passed [74] for all the factors. The KMO index is a measure of sampling adequacy that ranges from 0 to 1, considering a value greater than 0.5 suitable for factor analysis. Bartlett’s Test of Sphericity should be significant (*p* < 0.05) for factor analysis to be suitable, which occurred for all the factors. Table 2 shows that, in seven of the nine factors, the explained variance exceeded 60%, and in the other two factors, it was above 50%. Factor loadings presented values greater than 0.7, except in the case of two items that had values greater than 0.67. Regarding the reliability of the factors considered, Cronbach’s alpha coefficient exceeded 0.7 in almost all cases, with the exception of the factors that measured social and lifestyle motivations.

### 3.2. Types of VCS Used by Farmers Engaged in On-Farm Diversification

The first research question concerned the types of VCS used by farmers engaged in diversification initiatives. Table 3 presents the proposed structure and the variables used in this study for each dimension of the VCS. 

Using cluster analysis, four types of VCS were identified in order to study their differing characteristics and performance. We used the K-means technique to carry out the cluster analysis, using variables from Table 3. To avoid overweighting certain factors, variables presenting over 40% correlation with other variables were not considered, as was the case for ‘Knowledge management’, ‘Participative management’, and ‘% of sales to large-scale distribution’. In this way, the remaining seven variables in Table 3 could be used to perform the cluster analysis. However, we tried to find a balance between the sample size and the variables considered. Given that in some previous work [76] the proposed minimum size of the sample to perform the cluster analysis was equal to 2^k^, with k being the number of variables to be used in the classification, we decided to use five variables (2^5^ = 32 < 49) instead of seven to perform the cluster analysis, which included number of product references, sales of fresh milk, sales of PDO, sales of organic products, and product innovation; the variables were standardised [74]. We decided not to use value proposition and networks because in the initial analysis they did not present significant differences between the groups (in the case of value proposition), or the difference was significant at a 10% level (in the case of networks). 

In order to determine the number of groups to be obtained with the cluster analysis, we applied the silhouette coefficient, which is a measure of both cohesion and separation, and the Akaike information criterion; both measures supported the choice of four groups (see Appendix A). In line with [74], the number of clusters also was set based on the judgment of researchers, which was formed from the knowledge acquired about the sector in the interviews with farmers and experts. In this sense, for the dairy diversification initiatives, we observed the existence of cases that worked with certified production through labels such as organic production and PDO, compared to others that were based on the transformation of conventional milk. There were cases in which only fresh milk was marketed, whilst in others the production of more elaborate products such as cheeses, yogurts, and other products was addressed. This reality led us to consider four different groups.

In Table 4 the four VCS are described, and the differences between them were analysed using the Kruskal–Wallis test and Dunn’s test. Owing to the reduced size of the sample and the cluster groups, and with a view to avoid distortions created by extreme values, Table 4, Table 5, Table 6, Table 7 include median values (except for the percentages, where the average was used as it is considered more appropriate for variables limited to values between 0 and 100%). Taking into account the values shown in Table 4 for the variables used in the cluster analysis, we labelled the four VCS as Ecological, Single-product, Innovative, and Traditional.

#### 3.2.1. Ecological VCS

These ventures are based on organic farms with a high degree of orientation towards the environmental dimension. Direct sales represented 41.4% of total sales, proving to be the VCS with most sales outside the region and with the greatest weight of yogurt in its production mix. The farms of this VCS stood out because of their high motivation towards a direct relationship with the final consumer and, with this objective in mind, the development of initiatives to a much greater extent than the other VCS. Thus, in this VCS 63.4% of the farms adopted initiatives that favoured direct communication with the customer, such as agro-tourism, farm interpretation centres, guided tours of farms, museums, shops on the farm, etc., as represented by the variable relational diversification. 

The Ecological VCS is characterised by social motivations rather than seeking high-growth business opportunities. This VCS also had a high level of participative management, implying that employees were involved and committed in the decision-making process. In our sample, the Ecological VCS also presented the highest percentage of managers with university studies (54.5%).

#### 3.2.2. Single-product VCS 

The Single-product VCS is characterised by its focus on fresh milk sales (95.4% of total sales). The majority of farms of this cluster maintained vending machines as the principal channel for commercialising fresh milk. These farms had a low level of certification (PDO, organic). They had a high percentage of direct sales, which requires a bigger marketing effort, observable in the form of a larger number of workers and investments dedicated to these activities, reflected in a greater weight of fixed costs for commercialisation purposes. These farms are based on a single product and offer a reduced number of product references. 

This VCS consisted of farms with high milk production (820,000 litres), but the milk processed by the value-added venture represented just over 20% of overall production. In general, the milk production of these farms is based on intensive systems, characterised by highly productive cows, and a great dependence on both external inputs (feed, fertilisers, machinery, etc.) and the dairy industry. The farms in this VCS only used cow’s milk, coming in almost 90% of the cases from Friesian cows. This contrasted with the other three VCS, where milk from goats and sheep was used to a greater or lesser degree together with that of other cow breeds, generally more rustic and adapted to the local environment, such as Galician Blond or Brown Swiss, which provide a differential quality to products, both physical (fat, protein) and intangible (image).

The Single-product VCS comes with a high economic motivation, which tries to add value to milk production and seeks to reduce the great dependence on the dairy industry. Due to this, diversification in these farms, especially over recent years, tends to be dependent on push factors (price reduction, low bargaining power, etc.). The diversification is opportunity driven (“pull factor”) when new activities are started because the farmer has seen a business opportunity. The diversification is necessity driven (“push factor”) when the farmer has to diversify in order to secure family income or decrease risks caused by changes in the market situation [77].

In general, according to their narratives, these farmers trust in the quality of their primary production, and in their technical skills and management capacity. However, they face difficulties in differentiating themselves from the dairy industry due to, among other reasons, the minimal transformation of production (they sell fresh milk), a narrow range of product references, as well as their defensive strategy (61.9% of the cases).

#### 3.2.3. Innovative VCS 

These cases display a high degree of innovative behaviour. Their production featured a low level of product certification (PDO, organic), with cheese representing their most important product (61.1% of sales). This VCS had the highest number of product references due to an innovative effort aimed at encompassing different segments of interest.

The innovative VCS showed the highest value of milk processed (71% of total milk produced). In some cases, this percentage exceeded 100%, requiring the purchase of milk from outside the farm to cover demand.

Although this VCS presented a low level of products certified (PDO, organic label), farmers are able to use the farm’s resources and features in flexible and innovative ways to create value [78].

These farmers are motivated by pull factors and are very oriented to the search of business opportunities, most of these ventures being run by prospectors and analysers. These ventures are innovative and proactive, seeking new market opportunities. 

Taking into account what was stated by the interviewees in relation to new projects, as a reflection of the EO of these ventures, several initiatives can be cited that try to take advantage of different market opportunities: increase in the product range, different product sizes, innovation in packaging, benefiting from regional tourism revitalisation plans, use of their own distribution to market other agricultural products, establishment of their own stores in cities for product sales, participation in benchmarking and innovation projects, etc.

#### 3.2.4. Traditional VCS 

These initiatives are older than those of other VCS, being dedicated to the production of traditional cheese well-known in the market due to PDO protection. Since the PDO label is an instrument that reduces the asymmetric information problem between producers and consumers, if the collective reputation of the product is good, this label will be a powerful tool for signalling quality [79]. This has allowed these initiatives to sell a significant part of their production via large-scale distribution, which in turn encourages sales outside the region of origin. These cases have a low level of innovative behaviour and are specialised in traditional products recognised through a PDO. 

Many of these farms produce cow, goat, and/or sheep milk and possess good technical skills in relation to the production of traditional cheeses, as they have extensive experience in dealing with consumers through participation in local markets.

Most farms in the Traditional VCS develop defensive strategies, focusing on traditional products and established markets. They have a low level of participative management. 

### 3.3. Contexts and Entrepreneurial Capabilities Associated with VCS

**Figure 1:**
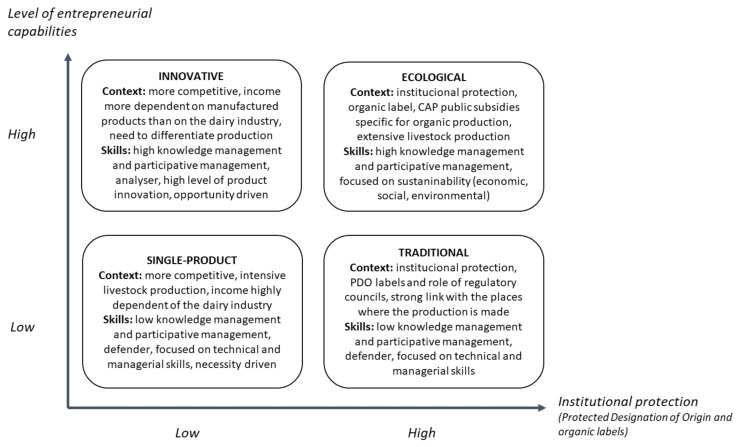
Matrix context and entrepreneurial capabilities.

Since the business model considers the key resources and activities for developing the value proposition delivered to consumers, it is possible to highlight the entrepreneurial capabilities involved and the context of each VCS, both summarised in Figure 1.

Based on the analysis presented in previous sections, we have identified four VCS within the value-added initiatives. This can be illustrated graphically via two dimensions related, on the one hand, to the type of context and, on the other, to the level of entrepreneurial skills of the farmers. Figure 1 graphically displays a matrix where the *x*-axis reflects the context as the level of institutional protection, while the *y*-axis shows the level of entrepreneurial capabilities.

In the four VCS, we observed different contexts determined by factors such as regional location, dependence on the dairy industry, the level of compliance with the requirements to be able to use a quality certification as a PDO or the organic label, and the level of intensification of the farm’s productive system. Given that the context incorporates multiple dimensions, such as business, social, spatial, and institutional [34], in order to facilitate a graphic representation (Figure 1), the level of institutional protection is considered as the context indicator. We assume that initiatives based on the use of labels (PDO, organic) benefit from institutional protection that allows them to reduce the competitive pressure to which the dairy sector is subjected. 

Furthermore, our analysis describes different levels of entrepreneurial skills or capabilities between the four VCS. We show VCS as Single-product or Traditional, where technical and managerial skills tend to predominate, in contrast with Ecological and, especially, Innovative VCS, characterised by a high level of entrepreneurial skills.

The use of the Miles and Snow typology allowed us to observe how the defensive character predominated in the Single-product and Traditional VCS, which is related to a low proactive character and, ultimately, to a low level of entrepreneurial skills [71,80]. Traditional and Single-product VCS corresponded to the dairy farms with the highest production (Table 5), and in these cases, especially in the Single-product VCS, most farmers saw their activity as product-oriented, focused on competing in terms of cost [81]. Table 6 shows a greater weight of the factors that represent knowledge management and participative management in Ecological and Innovative VCS, which suggests the importance of these capabilities for the identification and exploitation of new opportunities. Although not all entrepreneurial skills are amenable to teach [36,82], their learning can be facilitated if the participation of all the workers in the management of value-added initiatives is enhanced.

It is also observed that, in similar contexts, the personal characteristics of the entrepreneur determine whether the VCS adopted is different. In this sense, there are ventures that, due to the spatial context in which they operate, could belong to the Traditional VCS but, due to the farmer’s EO, fit better into the Innovative VCS. Likewise, farms that could be certified as organic are not and remain in the Traditional VCS because the entrepreneur prefers to continue with a defensive strategy and does not wish to affront changes in their productive system.

### 3.4. Comparison of Performance between VCS

The third research question explored differences in performance between VCS. Table 7 presents the performance values for the four VCS.

According to the Kruskal–Wallis test, significant differences only existed for the variable margin per litre. Based on the results of Dunn’s test, the Ecological and Innovative VCS presented a greater margin per litre than the Single-product VCS, just as the Ecological also surpassed the Traditional. Nevertheless, no significant differences were found for the profit per litre, value-added per worker, and qualitative performance. 

The Single-product VCS corresponded to younger ventures focused on fresh milk, many of which were created from 2009 onwards for the distribution of milk to consumers through vending machines. The evolution from this VCS to others with greater value-added products and the use of different channels requires more entrepreneurial skills. 

Our results indicate that it is possible to add value to primary production through various VCS and with different EO. For example, Traditional VCS farms generate value with a lower level of EO because they meet the requirements for benefiting from the advantages of the PDO certification. On the other hand, Innovative VCS farms that mostly work with conventional milk and without the protection of a PDO or the organic label operate with the highest levels of EO in order to be successful in their economic activity.

## 4. Discussion

The growth of milk production experienced by Spain in recent years accompanied by a significant reduction in the number of farms has accentuated their specialisation, leading to more intensive production systems, with greater dependence on the dairy industry and external purchases. These types of specialised farms present a high market risk, being very vulnerable to changes in milk prices [9,23]. Value-added diversification strategies seek to provide income to farmers, reducing the risk presented by specialisation strategies, as well as contributing to the other objectives of farmers related to the generation of family employment and lifestyle. These types of strategies lead farmers to use underexploited agricultural resources, strengthening farm resilience and favouring the diversification of rural economies [21]. The implementation of these types of initiatives and their promotion by public administrations require empirical evidence on the different VCS, their internal and external contexts, and their effect on economic results.

This study contributes to the field of agricultural entrepreneurship and farm diversification by uncovering the heterogeneity of the value-added strategies followed by dairy farmers in a changing environment and revealing how they seem to align their capabilities and contexts. To do this we adapt an investigative framework based on the BMC that shows the informal strategies followed by dairy farmers in their value-added ventures [39]. Since the canvas model considers the key resources and activities required to develop the value proposition transmitted to consumers via different products, quality schemes, mixtures of milk from different animals (cows, goats, and sheep) and breeds, sales formats, and distribution channels, it is possible to highlight the context and entrepreneurial capabilities of each of the VCS. Hence, this paper also answers the call for contextualising entrepreneurship research [34,43], showing how the context combined with the personal characteristics of the entrepreneur both enable and constrain the entrepreneurial behaviour of the farmers [83]. Moreover, this paper complements the results obtained by [33], highlighting the existence of heterogeneity in value-added initiatives of the dairy sector and showing significant differences in the extent to which farmers make use of local resource bases.

We used quantitative and qualitative data from 49 Spanish dairy farms involved in diversification via the processing and marketing of dairy products (fresh milk, cheese, yogurts, and others). Our database offers suitable material in order to address the research questions posed. First, although all farmers are in the dairy sector and they elaborate similar products, the granularity of the data allows us to describe the features of their different VCS. The in-depth interviews have allowed us to complement the study with narrative elements important in strategic formulation and in context analysis. The combination of the farmers’ comments and the experience of the researchers helped to properly interpret the results obtained. Second, because the production and distribution costs linked to the activities of the processing and marketing of the dairy products are available, we can measure the real contribution of the value-added activities to the farm’s financial performance. While the collection of qualitative data allows us to capture the richness and diversity of the context, the use of quantitative methods offers legitimacy to our results [34]. In this sense, our study makes a significant contribution to the literature regarding the effect of diversification on farm results, overcoming one of the limitations posed by previous studies that lack the information necessary to adequately separate the income and costs corresponding to diversification initiatives [23,84]. 

Furthermore, this paper provides a worthwhile lesson by underlining the importance of developing management accounting systems in the context of farm diversification initiatives. The systematic collection of variables such as those used in this work permits the construction of datasets, currently unavailable, that allow a systematic evaluation of the effectiveness of diversification initiatives. These also represent an important source of information for monitoring the political measures proposed in the farm-to-fork strategy [2]. Although our dataset is not recent, and we should be cautious about policy recommendations, it does allow us to uncover several factors that are related to the different VCS, and it will be interesting to see whether and how these relationships evolve over time and how they compare to experiences in other places. As such, we consider that the present study provides a point of departure for an important potential line of research on the issue of sustainability in small-scale agricultural production. We hope that our work will encourage researchers to carry out similar studies in other regions and countries.

Our study shows that there are contexts where success can be achieved with a weaker EO due to greater institutional protection provided by labels such as PDO (e.g., Traditional VCS). In other cases, where the conditions to take advantage of institutional protection are not fulfilled, acting with a greater EO is essential in order to differentiate products in the market and ultimately achieve success (e.g., Innovative VCS). The Single-product VCS showcases the risks and problems of operating in the market without institutional protection (via PDO certification or organic label) and with a weak EO.

It should be borne in mind that barriers to entry and to mobility exist when operating with the Ecological, Innovative, and Traditional VCS. Hence, the Ecological VCS requires a milk production certification on the farm in accordance with the regulation established by the European Union. The aforementioned certification for a PDO is associated with certain geographical regions and production standards, whilst the Innovative VCS requires time for the adequate establishment of networks, product innovation, and a market presence through various channels with new product references. 

Our study reveals interesting results related to how value is produced and how it is apparently related to the farmer’s own values. In our sample, most of the farmers from Single-product and Traditional VCS expressed that they consider their future dependent on the high quality of their products. Nevertheless, they seem to pay scarce attention to the management of the intangible and symbolic aspects of quality agri-food products. In these cases, a reduced level of entrepreneurial skills was observed, with attention focused on a single resource (the physical quality of the products). Precisely, the most specialised farms, with the highest production volume, corresponding to the Traditional VCS and especially the Single-Product VCS, were those that have a strong focus on production techniques (engineering approach), trying to improve labour productivity, while the Ecological and Innovative VCS presented a greater EO, assigning more importance to a marketing approach [9].

With reference to the engagement in socio-material aspects, our typology reflects how farmers respond to the challenges and potential of rural activity in a diverse manner [85]. The resources available to farmers in rural areas (material, natural, social, cultural, heritage) can be used and recombined creatively to take advantage of new entrepreneurial opportunities and create value in different ways. This process of resource recombination adopts material resources as a basis and is leveraged by different degrees of those intangible resources, characteristic of rural communities. Notably, in Ecological and Innovative VCS, farmers exploit intangible resources that provide additional value to product quality. Normally, organic farms have an extensive productive system based largely on pastures, showing great motivation for maintaining the landscape and the rural environment, which on many occasions leads them to the development of value-added initiatives based on products and services that try to provide unique experiences to the final consumers. These farmers have a high level of placial embeddedness taking into account ‘experiential and meaning-related dimensions such as culture, heritage, and history’ [86], which serve to favour direct communication with the customer (agro-tourism, guided tours to farms, etc.). They can follow strategies based on face-to-face links with consumers, in which authenticity and trust are mediated through personal interaction [87]. In accordance with [88], we confirm that peripheral spaces, where most of the farms we analysed were located, are becoming areas of consumption where immaterial factors linked to the territory have greater relevance than material elements, thereby generating new opportunities for entrepreneurs related to experiential consumption. The entrepreneurs of the different VCS differ in the capacity to recognise and take advantage of these opportunities. 

Moreover, in line with more recent research [33], different levels of local resource embeddedness and spatial bridging are observed in the four identified VCS. On the one hand, in the VCS with more entrepreneurial skills (Ecological and Innovative), a higher level of local resource embeddedness is present, highlighting greater creativity in the use of these resources. Traditional VCS also has a high level of local embeddedness, due to the use of PDO certifications based on specific cheeses linked to a specific geographical origin as well as traditional know-how. The cases that integrate Single-product VCS are located in dairy farms with intensive production systems that are highly dependent on inputs external to the farm and the region of origin (fertilisers, feedstuffs and others). On the other hand, there is a process of codification which makes the chosen combination of resources transferable and comprehensible to consumers, who are mostly located in urban areas. This codification takes different forms in the ventures analysed, ranging from the creation and promotion of private brands to the use of collective certifications. Product qualification is a mechanism for linking local farmers and non-local actors by which farmers can signal to, and attract revenues from, exogenous actors [89]. In this context, the use of labels such as PDO and organic production facilitates a higher level of bridging with geographical areas other than the region of origin, as observed by the higher percentage of sales outside the latter region in the cases of Traditional and Ecological VCS. The minimum percentage of sales outside the region stands out in the case of the Single-product VCS.

In particular, in the case of the Single-product VCS, integrated by recent initiatives, despite weighing a high value on the product innovation factor, a low level of entrepreneurial skills is observed. This is related, among other things, to the low score in knowledge and participative management factors, the predominant character of the defensive strategy, and the reduced consideration of intangible assets. In the years prior to 2011, the reduction in the price received for milk sales to the dairy industry pushed a large portion of the farmers belonging to the Single-product VCS towards diversification in an attempt to maintain their income level, this despite a low level of entrepreneurial skills.

Consequently, in an increasingly open and dynamic market, farms in the Single-product and Traditional VCS should try to create value strategies that enhance their market orientation. New products and product characteristics, services, brand names, or unique customer experiences may create additional value for farms. A suitable combination of tradition and innovation could be the source of greater business initiatives with very positive effects for regional development. In this process of the recombination of resources, there are farmers who contribute more than others to enhancing the value of the rural area, becoming agents of rural change, leveraging on spatial resource endowment and spatial bridging [33]. These types of initiatives require more complex skills and capabilities than those associated with traditional production. In this sense, [90] points out the importance of encouraging innovation and entrepreneurship in PDO farms in order to improve the sustainability of rural areas. 

The development of skills for the implementation and management of diversification initiatives is a relevant issue, which should be treated appropriately based on the age and previous experience of the farmers, taking into account that older farmers could show greater resistance to change [18]. Due to the range of skills and knowledge required for roles involved in entrepreneurship, an appropriate combination of theory and practice is required for the development of entrepreneurial competence [82]. Hence, measures that facilitate coaching and mentoring of agricultural entrepreneurs would seem appropriate [91].

We understand that this work makes an important contribution to political decision makers, farm advisers, and farmers, by highlighting heterogeneity in VCS of diversification initiatives, which has made it possible to observe that different solutions and measures are required depending on the contexts and entrepreneurial skills present in each case. This has clear implications for guiding decision-making processes and public subsidies in order to improve the sustainability of farms.

The use of dairy cows of more rustic breeds and adapted to the local environment, such as Galician Blond or Brown Swiss, provides tangible (more fat and protein) and intangible (image) elements that contribute to the differentiation of the value-added strategies followed by dairy farms. In this sense, the diversification ventures of dairy farms prove an appropriate element for guaranteeing the survival of certain local breeds as well as making their use profitable.

## 5. Conclusions

This paper is focused on milk production, a traditional sector of European farming, which is currently facing major changes. We study the incorporation of value added to the agricultural product through food processing and direct marketing, a type of diversification promoted by the EU and supported by the CAP [45]. Using the data of 49 dairy farms located in Northern Spain, which elaborate and sell dairy products, four Value-Creating Strategies (VCS) were identified and denoted as Ecological, Single-product, Innovative, and Traditional. This study contributes to clarifying the relationship between VCS, the entrepreneurial capabilities, and the context, which is important for farmers and policymakers because it reveals the diversity of farm management and the resilience of farm systems.

The VCS are associated with contextual factors such as the region, compliance with the conditions for producing with a label of quality (PDO, Organic), and the level of intensification of the farm productive system. 

The characteristics of the entrepreneur and their motivations also influence how the entrepreneurial activity is carried out, in such a way that farms in similar contexts and with different entrepreneurial skills and motivations opt for different VCS. For example, some cases, due to their location, could belong to the Traditional VCS but, due to the level of entrepreneurial capabilities, instead belong to the Innovative VCS.

In relation with the performance of each VCS, results reveal that there are no significant differences between the VCS in terms of profit per litre, value added per worker, and qualitative performance, while the Ecological and Innovative groups present the highest margins per litre. However, a more open market means potential challenges for Single-product VCS and Traditional VCS, which could be tackled with the incorporation of greater entrepreneurial skills.

In terms of implications for practice, our results are useful not only for those farmers planning these types of diversification initiatives but also for the public administrations interested in promoting them. The analysis suggests that policymakers should consider the heterogeneity of agrarian diversification initiatives, given that the various VCS in turn exert differing impacts on the development of rural areas. Our typology of ventures could be useful as a starting point for discussions concerning how to support entrepreneurship in farms through policy measures [85]. In this sense, we agree with [81] in that the development and continuity of agricultural activity require political measures aimed at favouring the education and training of those entrepreneurial skills essential for farmers if they are to survive and succeed in an increasingly competitive environment. 

Finally, this study presents several limitations, among which are the following: the small size of the sample, the availability of data for a single year only, and lack of information about the farmer’s family characteristics. Future work is required to analyse the evolution of the VCS for farm diversification over multiple years, as well as their possible effect on performance. In particular, since the VCS are not static, a detailed examination of their temporal evolution proves of great interest. Additionally, gathering more information regarding the characteristics of the farmer and his/her family is essential for analysing the role played by these in the VCS to be applied in agricultural diversification [40]. The latter serves to observe and explain the effects of each context and the farmers’ personal characteristics when changing from one VCS to another as well as the repercussions for performance. 

## Figures and Tables

**Table 1:** Descriptive statistics of the 49 initiatives.

Variable	Mean	Std. Dev.	Min.	Max.
Processed litres per venture	238,548	492,421	1000	3,443,856
Investment (EUR)	257,500	200,095	0	991,180
Number of workers	3.1	2.4	0.4	14.0
Sales (EUR)	256,808	310,996	16,076	1,974,490
Sales of yogurt ^a^ (%)	11.3	21.9	0	91.4
Sales of cheese ^a^ (%)	54.1	45.7	0	100
Sales of fresh milk ^a^ (%)	22.5	36.9	0	100
Sales of other products ^a^ (%)	12.1	30.4	0	100
Direct sales ^b^ (%)	35.2	25.5	0	95.0
Hospitality sales ^b^ (%)	21.0	19.7	0	100
Shop sales ^b^ (%)	30.0	26.3	0	100
Large-scale distribution sales ^b^ (%)	13.8	24.3	0	93.4
Sales of organic products (%)	26.8	43.1	0	100
Sales of products with PDO (%)	22.1	38.6	0	100
Cow’s milk in products (%)	87.2	27.0	0	100

^a^ Products are classified in four broad categories: fresh milk, yogurt, cheese, and others. ^b^ There are four sales channels considered: direct to final consumers, hospitality, shops, and large-scale distribution.

**Table 2:** Constructs used in the analysis.

Factor	Items (from 1 to 5)	Factor Loadings	Statistics and Tests	Previous Studies
Factors used for characterising the business model				
Value proposition	Ability to make quality products Responsibility of the company with the environment Customer satisfaction	0.899 0.766 0.781	Cronbach Alpha: 0.727 Factorial: 1 factor Explained variance: 66.8% Sig. Bartlett: 0.000 KMO: 0.599	[44,45]
Knowledge management	Workers propose innovations in the tasks related to production and processing Workers benefit from continuous learning dynamics Workers share new knowledge with colleagues	0.865 0.933 0.888	Cronbach Alpha: 0.872 Factorial: 1 factor Explained variance: 80.2% Sig. Bartlett: 0.000 KMO: 0.703	[46,47]
Participative management	Workers participate in the economic performance of the company Employees participate in making important decisions about business management There are mechanisms to collect, study, and implement the proposals of the workers for improving processes and products	0.829 0.900 0.858	Cronbach Alpha: 0.828 Factorial: 1 factor Explained variance: 74.4% Sig. Bartlett: 0.000 KMO: 0.697	[48,49,50]
Product Innovation	The firm usually proposes product innovation It proposes packaging innovation It proposes innovation in the commercialisation of products	0.816 0.784 0.871	Cronbach Alpha: 0.755 Factorial: 1 factor Explained variance: 68.0% Sig. Bartlett: 0.000 KMO: 0.669	[51,52]
Networks	A working relationship is established with customers A working relationship is established with competitors A working relationship is established with other agri-food companies A working relationship is established with other entities (administrations, universities, foundations, etc.)	0.799 0.768 0.831 0.712	Cronbach Alpha: 0.782 Factorial: 1 factor Explained variance: 60.6% Sig. Bartlett: 0.000 KMO: 0.781	[37]
Performance	Optimisation of the investment in assets Competitiveness of the company Profitability of the company	0.791 0.875 0.813	Cronbach Alpha: 0.766 Factorial: 1 factor Explained variance: 68.4% Sig. Bartlett: 0.000 KMO: 0.668	[19,38]
	Factors used for identifying the motivation behind diversification			
Economic opportunity motivation	Obtaining additional income Exploiting a market opportunity Customer request for products	0.679 0.756 0.713	Cronbach Alpha: 0.523 Factorial: 1 factor Explained variance: 51,4% Sig. Bartlett: 0.027 KMO: 0.608	[14,16]
Social motivation	Promote farm succession Create family jobs Minimise the risk of farm closure	0.850 0.852 0.854	Cronbach Alpha: 0.819 Factorial: 1 factor Explained variance: 72.6% Sig. Bartlett: 0.000 KMO: 0.716	
Lifestyle motivation	Lifestyle Direct relationship with consumers Independence from the dairy industry	0.797 0.767 0.673	Cronbach Alpha: 0.610 Factorial: 1 factor Explained variance: 55.9% Sig. Bartlett: 0.002 KMO: 0.618	

**Table 3:** Dimensions and variables of the structure proposed for the analysis.

Dimensions	Variables
Value proposition	- Value proposition
Customer	- Sales of fresh milk (% of total sales)
	- Sales of products certified with protected designation of origin (PDO) (% of total sales)
	- Sales of organic products (% of total sales)
	- Sales to large-scale distribution (% of total sales)
	- Number of product references
Key activities	- Knowledge management - Participative management
	- Product innovation
	- Networks

**Table 4:** Variables used in cluster analysis to identify value-creating strategies.

Variables	K–W Test	Ecological (1)	Single-Product (2)	Innovative (3)	Traditional (4)	Dunn’s Test
No. firms		11	9	8	21	
Sales of fresh milk (%)	***	15.1	95.4	5.2	1.7	***: 1–2, 2–3, 2–4
Sales of products with PDO (%)	***	2.8	0.0	5.2	48.0	***: 2–4/**: 1–4
Sales of organic products (%)	***	99.3	13.2	4.0	3.2	***: 1–2, 1–3, 1–4
Product innovation	***	−0.120	0.621	1.526	−0.559	***: 2–4, 3–4
Number of references	***	5.00	3.00	11.50	4.00	***: 2–3, 3–4/**: 1–3

Notes: for the values expressed in percentages the mean is shown and the median for the rest. ***, **, indicate statistical significance at the 1%, and 5% level, respectively.

**Table 5:** Production and commercial characteristics of the value-creating strategies.

Variables	K–W Test	Ecological (1)	Single-Product (2)	Innovative (3)	Traditional (4)	Dunn’s Test
Production characteristics						
Venture age (years)	**	5.0	3.0	6.0	10.0	**: 2–4
Total litres produced in the farm	***	150,000	820,000	196,000	350,000	**: 1–2
Processed litres per venture		62,857	170,000	139,400	126,500	
Sales of dairy products (EUR)		129,340	166,908	249,756	196,620	
Number of workers per venture		2.0	2.0	3.0	3.0	
Investment (EUR)		179,180	244,736	262,918	176,574	
% Workers in processing ^a^	**	65.5	39.4	65.5	61.0	**: 1–2/*:2–3, 2–4
% Investment in processing ^a^	***	87.8	40.6	78.7	82.0	***: 1–2, 2–4/*:2–3
% Fixed costs of processing divided by total fixed costs ^a^	***	69.0	37.6	66.3	64.8	***: 1–2, 2–4/**:2–3
% University-educated workers		23.9	5.6	12.2	14.2	
% Family workers		71.5	45.6	72.7	53.3	
% Cow’s milk in products		91.0	100.0	77.7	83.4	
% of farms with all Friesian breed cows (dummy)		60.0	88.9	80.0	56.3	
% Female manager (dummy)		54.5	11.1	12.5	28.6	
Manager age (years)		42.0	47.0	39.5	45.0	
% Manager with university education (dummy)		54.5	11.1	12.5	28.6	
Commercial characteristics						
Direct sales ^b^ (%)	**	41.4	57.1	26.1	26.0	**: 2–4
Hospitality sales ^b^ (%)	**	11.8	29.1	31.4	18.4	*: 1–2, 1–3
Shop sales ^b^ (%)		37.9	13.7	31.5	32.2	
Large-scale distribution sales ^b^ (%)	**	9.0	0.0	11.0	23.3	**: 2–4
Sales of yogurt ^c^ (%)	**	23.8	2.5	15.8	6.8	**: 1–4/*: 1–2
Sales of cheese ^c^ (%)	***	36.5	2.1	61.1	83.0	***: 2–4/**: 1–4
Sales of fresh milk ^c^ (%)	***	15.1	95.4	5.2	1.7	***: 1–2, 2–3, 2–4
Sales of other products ^c^ (%)		24.6	0.0	17.9	8.5	
Number of products	**	2.0	1.0	1.5	1.0	***: 1–4
Number of references per product	***	3.0	2.5	6.8	4.0	***: 1–3, 2–3/*: 3–4
Sales outside the region (%)	**	24.1	1.1	13.1	15.2	**: 2–4
Relational diversification ^d^ (%) (dummy)	**	63.6	0.0	25.0	33.3	***: 1–2/*:1–3, 1–4

Notes: for the values expressed in percentages the mean is shown and the median for the rest. ***, **,*, indicate statistical significance at the 1%, 5%, and 10% level, respectively. ^a^ Workers, investments, and fixed costs were classified according to the transformation and marketing activities. The weights corresponding to transformation activities are shown, the rest being up to 100% relative to commercialisation. ^b^ Four sales channels are considered: direct to final consumers, hospitality, shops, and large-scale distribution. ^c^ Products are classified in four broad categories: fresh milk, yogurt, cheese, and others. ^d^ Relational diversification: dummy variable with a value of 1 when the farm undertakes initiatives that favour direct communication with the customer (agro-tourism, farm interpretation centres, guided tours to farms, museums, shops on the farm, etc.) and 0 in other cases.

**Table 6:** Motivations and other entrepreneurial characteristics of the value-creating strategies.

Variables	K–W Test	Ecological (1)	Single-Product (2)	Innovative (3)	Traditional (4)	Dunn’s Test
Economic opportunity motivation	*	−0.030	1.014	0.527	−0.439	*: 2–4
Social motivation	*	0.720	−0.045	0.201	−0.328	*: 1–4
Lifestyle motivation		0.476	0.563	0.003	−0.094	
Prospector (%) (dummy)		27.3	22.2	25.0	15.0	
Analyser (%) (dummy)		36.4	33.3	62.5	23.8	
Defender (%) (dummy)	*	36.4	44.4	12.5	61.9	**: 3–4
Knowledge management		0.654	−0.353	0.430	0.000	
Participative management	**	0.590	−0.107	0.463	−0.261	*: 1–4
Value proposition		0.974	0.105	−0.018	−0.300	
Networks		−0.557	0.211	0.685	−0.066	

Notes: for the values expressed in percentages the mean is shown and the median for the rest. **,*, indicate statistical significance at the 5%, and 10% level, respectively.

**Table 7:** Comparison of performance between value-creating strategies.

	K–W Test	Ecological (1)	Single-Product (2)	Innovative (3)	Traditional (4)	Dunn’s Test
Margin per litre ^a^ (EUR/l)	***	1.12	0.53	0.97	0.64	***: 1–2/**: 2–3/*: 1–4
Profit per litre ^b^ (EUR/l)		0.30	0.07	0.26	0.14	-
Value-added per worker ^c^ (EUR)		28,475	38,847	38,782	35,938	-
Qualitative performance		0.174	−0.705	0.133	−0.198	-

Notes: values shown are the median of the variables. ***, **,*, indicate statistical significance at the 1%, 5%, and 10% level, respectively. ^a^ Margin per litre: difference between income from the sale of dairy products and variable costs (milk, other raw materials, and packaging and labelling) divided by the number of litres processed. ^b^ Profit per litre: income from the sale of dairy products minus variable costs and fixed structural costs (personnel, depreciation, and general costs) divided by the litres processed. ^c^ Value-added per worker: total sales of dairy products, minus variable costs, and general costs of external services divided by the number of employees dedicated to processing and marketing.

**Figure A1:**
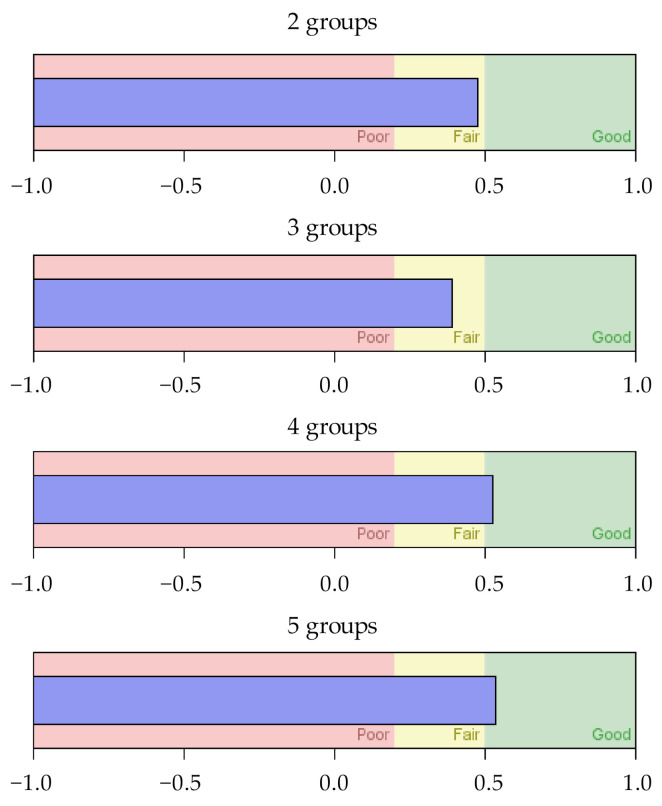
Silhouette measures of cohesion and separation.

**Figure A2:**
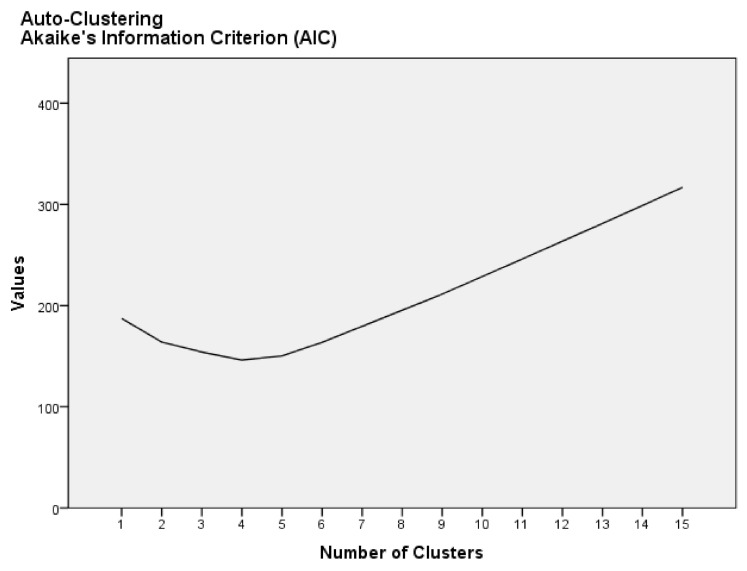
Akaike Information Criterion.

## Data Availability

Not applicable due to confidentiality clauses.

## Appendix A. Silhouette Measures and Akaike Information Criterion

The silhouette measure ranges from −1 to +1. In a good solution, the within-cluster distances are small and the between-cluster distances are large, resulting in a silhouette measure close to the maximum value of one. As can be seen in the graphs of the silhouette coefficient (Figure A1), the solution of four groups presented a higher value in this measure than the solutions of two or three clusters, with the indicator for five groups very similar to that of four. On the other hand, the Akaike criterion indicated the optimal solution with four groups.

**Table A1:** Akaike Information Criterion.

Auto-Clustering				
Number of Clusters	Akaike’s Information Criterion (AIC)	AIC Change ^a^	Ratio of AIC Changes ^b^	Ratio of Distance Measures ^c^
1	187.308			
2	163.931	−23.377	1.000	1.452
3	154.065	−9.867	0.422	1.073
4	146.226	−7.839	0.335	1.751
5	150.327	4.101	−0.175	2.416
6	163.746	13.419	−0.574	1.583
7	179.587	15.841	−0.678	1.000
8	195.429	15.841	−0.678	1.028
9	211.383	15.954	−0.682	1.521
10	228.722	17.339	−0.742	1.003
11	246.070	17.348	−0.742	1.088
12	263.631	17.561	−0.751	1.007
13	281.209	17.577	−0.752	1.060
14	298.922	17.714	−0.758	1.124
15	316.889	17.966	−0.769	1.080

^a^ The changes are from the previous number of clusters in the table. ^b^ The ratios of changes are relative to the change for the two-cluster solution. ^c^ The ratios of distance measures are based on the current number of clusters against the previous number of clusters.
